# Supplementary material for: Small Nucleolar RNA from S. cerevisiae Binds to Phosphatidylinositol 4,5-Bisphosphate
Source: Noncoding RNA. 2025 Jul 28;11(4):55. doi: 10.3390/ncrna11040055 (PMC12389098; doi:10.3390/ncrna11040055)
Supplement: Supplementary file 1 [file ncrna-11-00055-s001.zip › ncrna-3687694-supplementary.pdf]

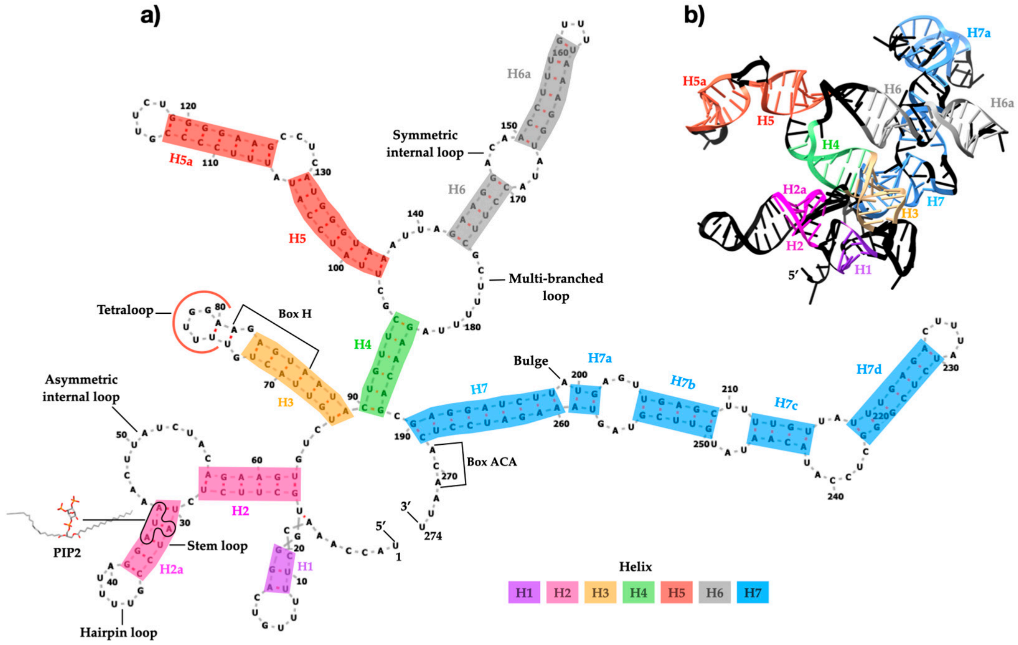


**Supplementary Figure S1**. Prediction of the 2D (panel a) and 3D structure (panel b) of snR191. The colors indicate the helices in both the 2D and 3D structures.

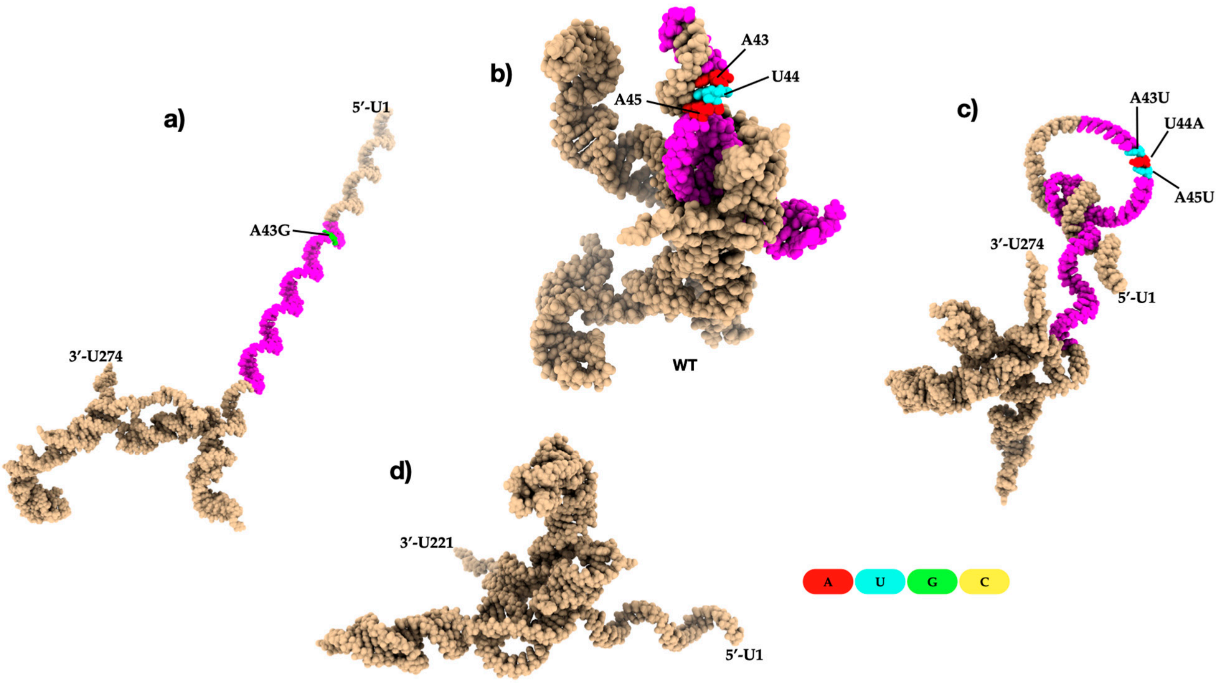


**Supplementary Figure S2**. Surface representation of the theoretical predictions of the mutations made in snR191 compared to the wild-type (WT) structure. Panel a, A43G mutation. Panel b, wild-type snR191. Panel c, triple mutations, A43U-U44A-A45U. Panel d, deletion of the 53-nt sequence. Magenta corresponds to the 53-nt region. Red is adenine, cyan is uracil, green is guard, and yellow is cytokinin.

**Table S1.** Identified motifs of snR191

| Sequence | Start | End | p-value | Protein | Motif |
|---|---|---|---|---|---|
| CUUUUUG | 8 | 14 | 0.045 | TIAL1 | 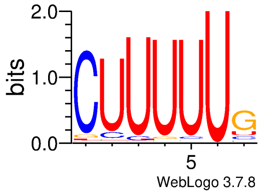 |
| gggggg[bbmmmm bb]gggggg | 66 | 87 | 0.026 | ABCF1 | 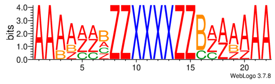 |
| UGUUUUG | 71 | 77 | 0.0091 | U2AF1 | 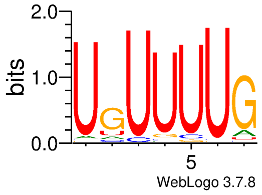 |
| GGAAGAG | 77 | 83 | 0.028 | APOBEC3C | 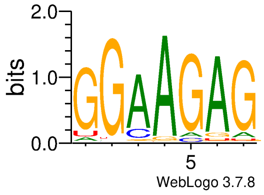 |
| hhhhhh[gggggggn nnnnggggggg4444 h | 99 | 129 | 0.038 | HNRNPUL1 | 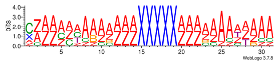 |
| CUGGGGAAGC | 116 | 125 | 0.048 | FXR2 | 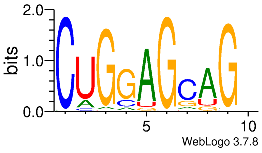 |
| GGGAAG | 119 | 124 | | PABPN1 | 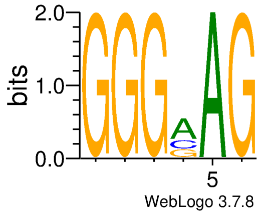 |

| | | | | | |
|---|---|---|---|---|---|
| AGAAGG | 140 | 145 | 0.039 | ZNF622 | 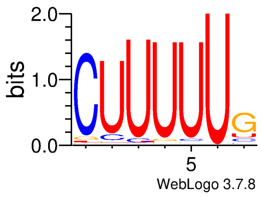 |
| CUUUGUUUU | 151 | 159 | 0.04 | FXR1 | 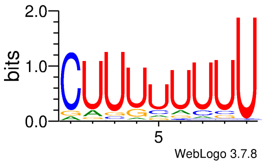 |
| UUGUUUUA | 153 | 160 | 0.012 | NIPBL | 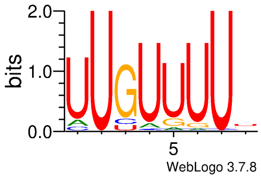 |
| UUGUUUU | 153 | 159 | 0.012 | TRA2A | 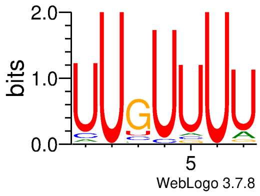 |
| UUUUAAA | 156 | 162 | 0.04 | U2AF2 | 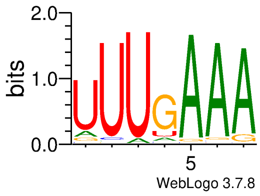 |
| UUUUAAA | 156 | 162 | 0.016 | TIA1 | 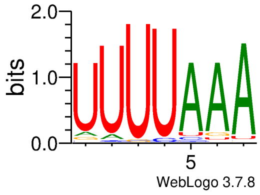 |
| UUUUGUUAUUU | 209 | 219 | 0.047 | TIA1 | 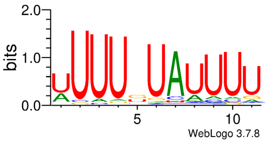 |

| | | | | | |
|---|---|---|---|---|---|
| UUUGUUAUUU | 210 | 219 | 0.025 | DDX6 | 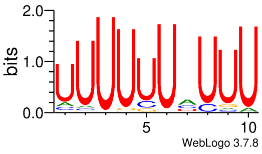 |
| UUGUUUU | 153 | 159 | 0.04 | AGO2 | 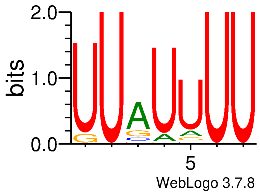 |
| UUUGAG | 217 | 222 | 0.039 | DGCR8 | 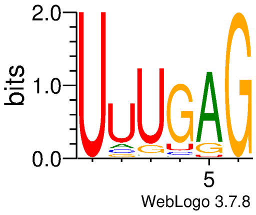 |
| UGUUUUAAA | 154 | 162 | 0.0067 | FMR1 | 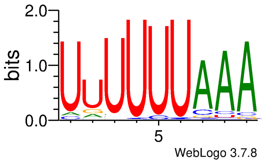 |
| UUUUGUUAUUU | 209 | 219 | 0.0099 | HuR | 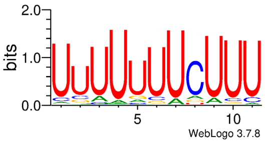 |
| UUUUAAA | 156 | 162 | 0.016 | EWSR1 | 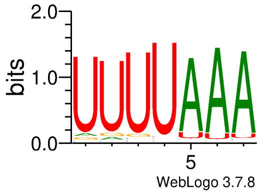 |
| CUUUGUUUU | 151 | 159 | 0.031 | PUM2 | 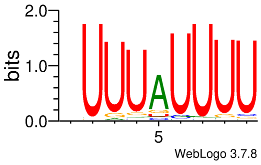 |

| | | | | | |
|---|---|---|---|---|---|
| UUUGUUUU | 152 | 159 | 0.042 | ZC3H7B | 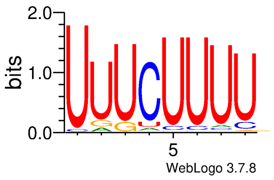 |
